# Accuracy of intraoral scanning in completely and partially edentulous maxillary and mandibular jaws: an in vitro analysis

**DOI:** 10.1007/s00784-020-03486-z

**Published:** 2020-08-19

**Authors:** Martin Schimmel, Norio Akino, Murali Srinivasan, Julia-Gabriela Wittneben, Burak Yilmaz, Samir Abou-Ayash

**Affiliations:** 1 Department of Reconstructive Dentistry and Gerodontology, School of Dental Medicine, University of Bern, Bern, Switzerland; 2 Division of Gerodontology and Removable Prosthodontics, University of Geneva, Geneva, Switzerland; 3 Department of Oral Implantology and Regenerative Dental Medicine, Tokyo Medical and Dental University, Tokyo, Japan; 4 Clinic for General, Special Care and Geriatric Dentistry, Center of Dental Medicine, University of Zurich, Zurich, Switzerland; 5 Division of Restorative and Prosthetic Dentistry, College of Dentistry, The Ohio State University, Columbus, OH USA; 6 Section for Digital Implant- and Reconstructive Dentistry [DIRecD], Department of Reconstructive Dentistry and Gerodontology, School of Dental Medicine, University of Bern, Bern, Switzerland

**Keywords:** Intraoral scanning, Digital impression, Scan time, Accuracy, Trueness, Precision

## Abstract

**Objectives:**

New generation intraoral scanners are promoted to be suitable for digital scans of long-span edentulous spaces and completely edentulous arches; however, the evidence is lacking. The current study evaluated the accuracy of intraoral scanning (IOS) in partially and completely edentulous arch models and analyzed the influence of operator experience on accuracy.

**Materials and methods:**

Four different resin models (completely and partially edentulous maxilla and mandible) were scanned, using a new generation IOS device (*n* = 20 each). Ten scans of each model were performed by an IOS-experienced and an inexperienced operator. An industrial high-precision scanner was employed to obtain reference scans. IOS files of each model-operator combination, their respective reference scan files (*n* = 10 each; total = 80), as well as the IOS files from each model generated by the same operator, were superimposed (*n* = 45; total = 360) to calculate trueness and precision. An ANOVA for mixed models and post hoc *t* tests for mixed models were used to assess group-wise differences (α = 0.05).

**Results:**

The median overall trueness and precision were 24.2 μm (IQR 20.7–27.4 μm) and 18.3 μm (IQR 14.4–22.1 μm), respectively. The scans of the inexperienced operator had significantly higher trueness in the edentulous mandibular model (*p* = 0.0001) and higher precision in the edentulous maxillary model (*p* = 0.0004).

**Conclusion:**

The accuracy of IOS for partially and completely edentulous arches in in vitro settings was high. Experience with IOS had small influence on the accuracy of the scans.

**Clinical relevance:**

IOS with the tested new generation intraoral scanner may be suitable for the fabrication of removable dentures regardless of clinician’s experience in IOS.

## Introduction

Digital technologies are increasingly used in daily life, which is a trend that can also be found in dentists’ clinical routine [1]. In dentistry, the introduction of the terms computer-aided design (CAD) and computer-aided manufacturing (CAM) marked the start of an unprecedented digitalization process. CAD-CAM procedures represent only one part of the digital processes, as they further comprise radiography, intraoral scanning (IOS), practice management, and patient recording, just to mention a few [2].

The IOS devices have evolved much and are currently available from a plethora of manufacturers since their first inception in dentistry in the 1980s [3]. Although with the technological advances the IOS devices now have higher accuracy, shorter scan times, and provide increased patient/clinician comfort, the basic principles of IOS still remain quite similar [4]. Consequently, digital scans for the fabrication of single- or short-span fixed partial dentures are a proven option today, with similar or even better outcomes regarding the accuracy and scan time, compared to conventional impression taking [5–9]. From a patient’s perspective, IOS appears to be more preferable to conventional impression taking in those scenarios, as it causes less discomfort [10]. Complete-arch scans in dentate sites have also been improving, and IOS can be successfully applied in those scenarios [11, 12]. However, in terms of accuracy, complete-arch scans still seem to remain inferior compared to conventional impressions [12, 13]. Furthermore, scan time may differ in different clinical complete-arch scenarios [14]. In a partially dentate scenario, the accuracy of IOS seems directly related to the size of the edentulous area, with higher inaccuracies when scanning extended edentulous areas [12, 15].

When it comes to removable partial (RPDs) or complete dentures (RCDs), it remains unclear whether IOS is a suitable option with regards to scan accuracy and scan time [16]. Nevertheless, complete digital workflows for the fabrication of RPDs and RCDs based on IOS data are available in the current literature [17–19]. The major challenge for taking intraoral scans in edentulous arches is the recording of the non-attached mucosa in the sense of a functional impression, as done in conventional workflows [20]. Due to the image-based nature, taking a functional impression with an IOS device is practically impossible, and the digital scans are taken under passive muco-static conditions [21]. However, clinical reports on IOS for the fabrication of RCDs and RPDs have reported clinically acceptable outcomes [17–19].

Recently introduced, new generation intraoral scanners are promoted as being suitable for scanning of extended or even completely edentulous ridges, even without reference markings, as suggested by some authors [22, 23]. The present study aimed to analyze the accuracy (trueness and precision) of IOS in completely and partially edentulous maxillary and mandibular models. The study further evaluated the influence of the operators’ experience with this new generation IOS device on the scan accuracy and scan time. The alternative hypothesis (H1) was that an IOS-experienced clinician would generate more accurate and faster scans compared to an inexperienced clinician.

## Materials and methods

### Study setting

**Fig. 1:**
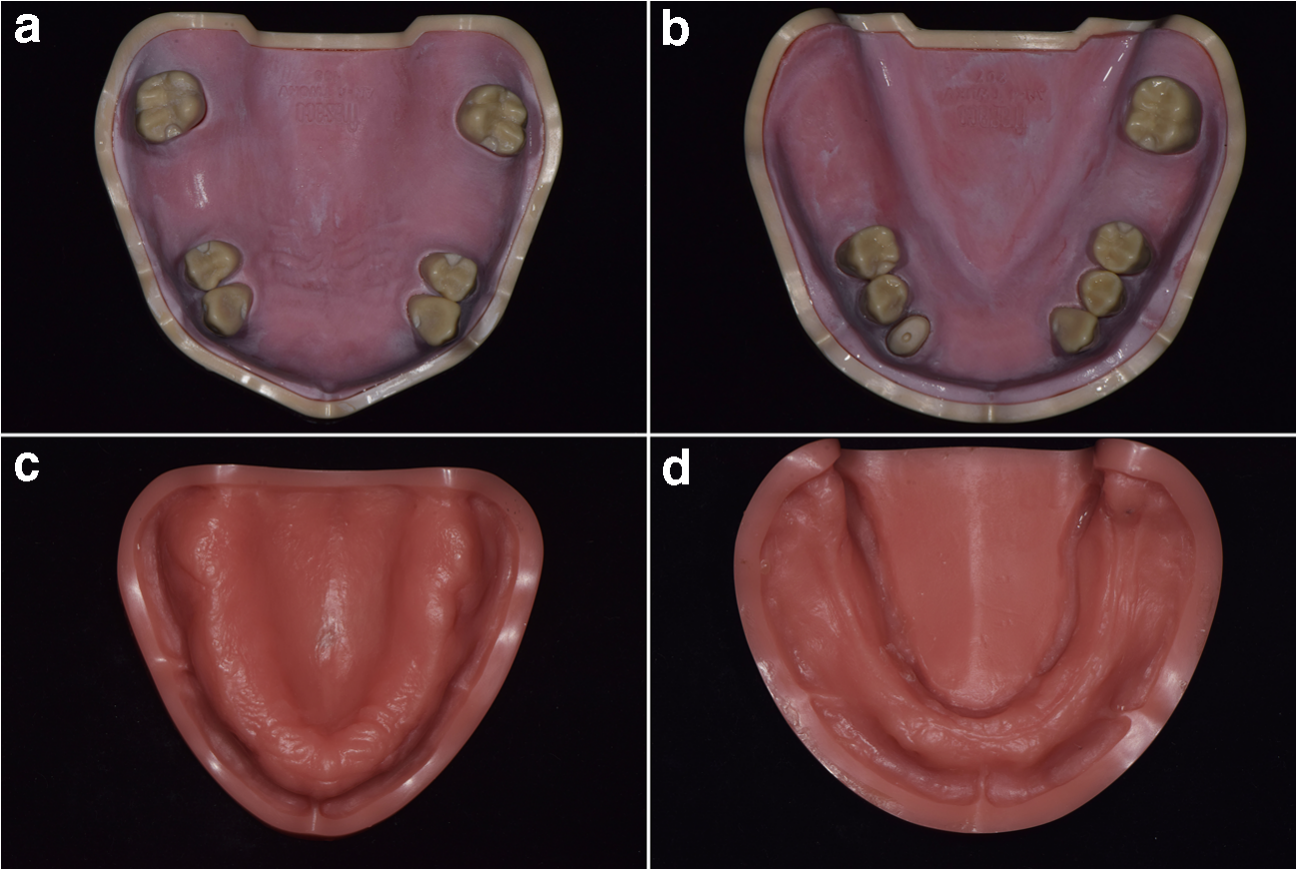
**a** Partially edentulous maxillary, **b** partially edentulous mandibular, **c** completely edentulous maxillary, and **d** completely edentulous mandibular model

Four different types of resin models, namely edentulous (B-3CSP; frasaco GmbH, Tettnang, Germany) and partially edentulous (ANKA-4; frasaco GmbH, Tettnang, Germany) mandibular and maxillary models (Fig. 1), were mounted on a phantom head (P-6/3; frasaco GmbH, Tettnang, Germany) with a face mask (P-6 GMN, frasaco GmbH, Tettnang, Germany) to simulate clinical conditions. The teeth in the partially edentulous models were prepared to receive a combined clasp- and attachment-retained (mandibular model, Kennedy Class II) or a clasp-retained RPD (maxillary model, Kennedy Class III). Digital scans were performed using a new generation IOS device (Primescan; Sirona, Bensheim, Germany) with the software version 5.0.2 by two specialist prosthodontists, one experienced and one inexperienced in IOS. Neither of the clinicians had ever used the tested IOS device before. Therefore, the manufacturer provided a theoretical instruction on how to use the device, explaining the technique and the recommended scan strategy. The two operators had no practical training before taking the simulated intraoral scans. All scans were made on the phantom head under dry conditions with ambient light. No information on the measuring uncertainty of the Primescan is provided by the manufacturer.

The decision on which type of model to start with was made by a coin flip, which was used to prevent the effect of “operator preference for scan order.” Both clinicians started with scanning the edentulous, followed by the partially dentate models (always: first maxillary-second mandibular model). Each operator took ten digital scans of each model (*n* = 10) resulting in a total of 20 scans per model and a total of 80 scans. The scan time of each scan was recorded separately, which included only the time for scanning, but not for subsequent software calculations. Afterwards, the scan data were exported in the standard tessellation language (STL) file format. For the reference data, all models were digitized using an industrial high-precision scanner (ATOS Capsule 200MV120; GOM GmbH, Braunschweig, Germany). Before the reference data were obtained, the calibration of the system was done by an independent calibration service (German Calibration Service – DKD) revealing a measuring uncertainty of 1 μm. The reference scan data were also exported in the STL format.

**Fig. 2:**
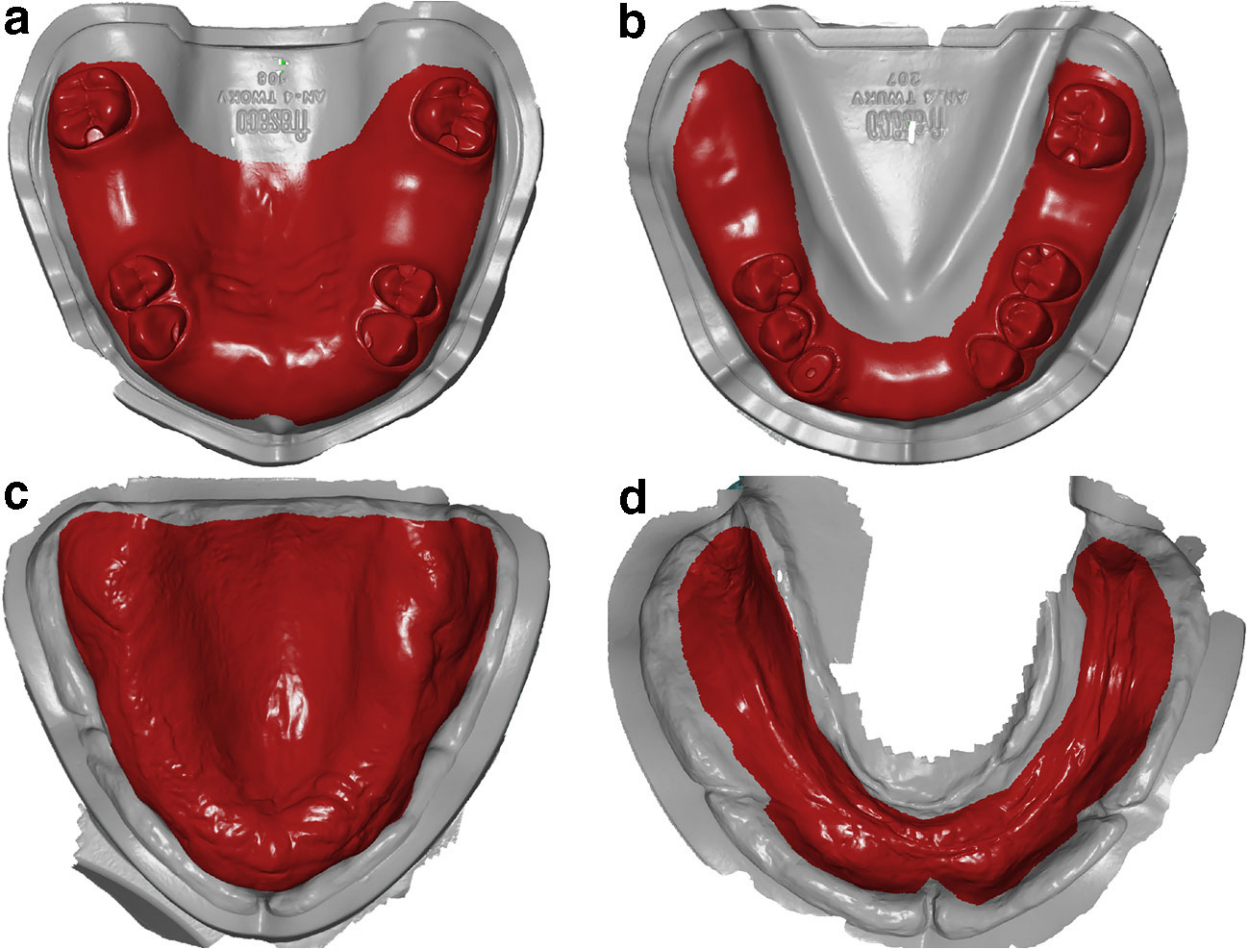
Region of interest digitally transferred from reference scan to a representative digital scan of each type of model. **a** Partially edentulous maxillary. **b** Partially edentulous mandibular. **c** Completely edentulous maxillary. **d** Completely edentulous mandibular model

Before starting the superimposition of the STL files, a region of interest (ROI), which represented the future extension of an RCD or an RPD was defined based on the reference STL files and was digitally transferred to the STL files obtained by IOS (Fig. 2). The prospective denture borders were marked to be approximately 2 mm away from the mucobuccal fold, resulting in denture border positions outside the area of the alveolar mucosa. Subsequently, the superimpositions were done with a software (GOM Inspect Professional; GOM GmbH, Braunschweig, Germany) applying a local best-fit alignment according to the respective ROI, using all surface points of the IOS data within this region. The number of those surface points was recorded for each scan. For trueness, the STL file of each model and operator was superimposed to the respective reference scan STL file (*n* = 10, *N* = 80). Afterwards, the average 3-D deviation using the absolute amount of the distances between all surface points of the IOS and the reference scan within the ROI was calculated [12]. For precision, all IOS data of the same model and operator were superimposed to each other (intragroup comparisons; *n* = 45, *N* = 360) and 3-D deviations were calculated the same way.

### Statistical analysis

For descriptive analyses, median values, interquartile ranges (IQRs), and minimum and maximum values were calculated. Trueness and precision were assessed in terms of the logarithm of absolute deviations (LAD), and the effect of the type of model and the operator were analyzed. For trueness, the impact of the factors “scan time” and “selected points” were additionally analyzed. The scan time was assessed in terms of the logarithm of scan time in minutes (LSTm).

Linear mixed models were used to model the LAD and LSTm. Thereby, the repeated scans were modeled as random values. An ANOVA for mixed models was used as an omnibus test to assess global differences, and a *t* test for mixed models was used to assess group-wise differences post hoc (for both types of tests, the Satterthwaite approximation was used). The impact of scan time and the number of surface points on LAD were assessed while correcting for the effects of model and executor (covariance analysis). Model accuracy was tested with the help of goodness-of-fit tests (Shapiro-Wilk) on residuals and random effects. *p* Values less than 0.05 were considered statistically significant. No corrections for *p* values were applied due to the explorative nature of this study. All statistical analyses were performed with using R software (version 3.5.0; R Development Core Team, https://www.r-project.org/, 2018).

## Results

**Fig. 3:**
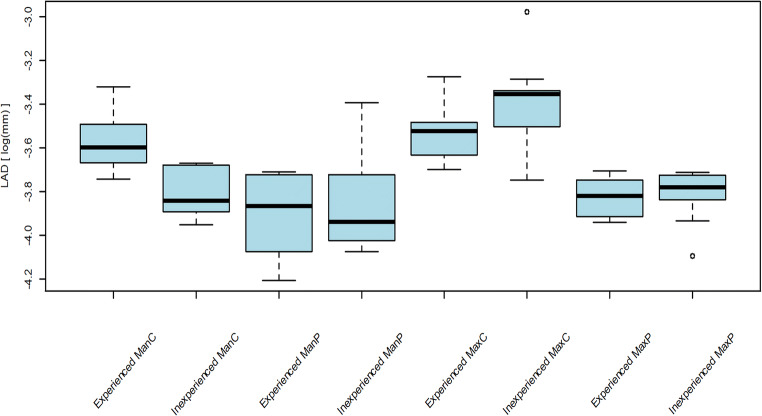
Trueness: logarithm of absolute deviations (LADs; *y*-axis), separated for two operators (experienced vs. inexperienced), and different types of models (ManC = mandible completely edentulous, ManP = mandible partially edentulous, MaxC = maxilla completely edentulous, maxilla = partially edentulous)

**Fig. 4:**
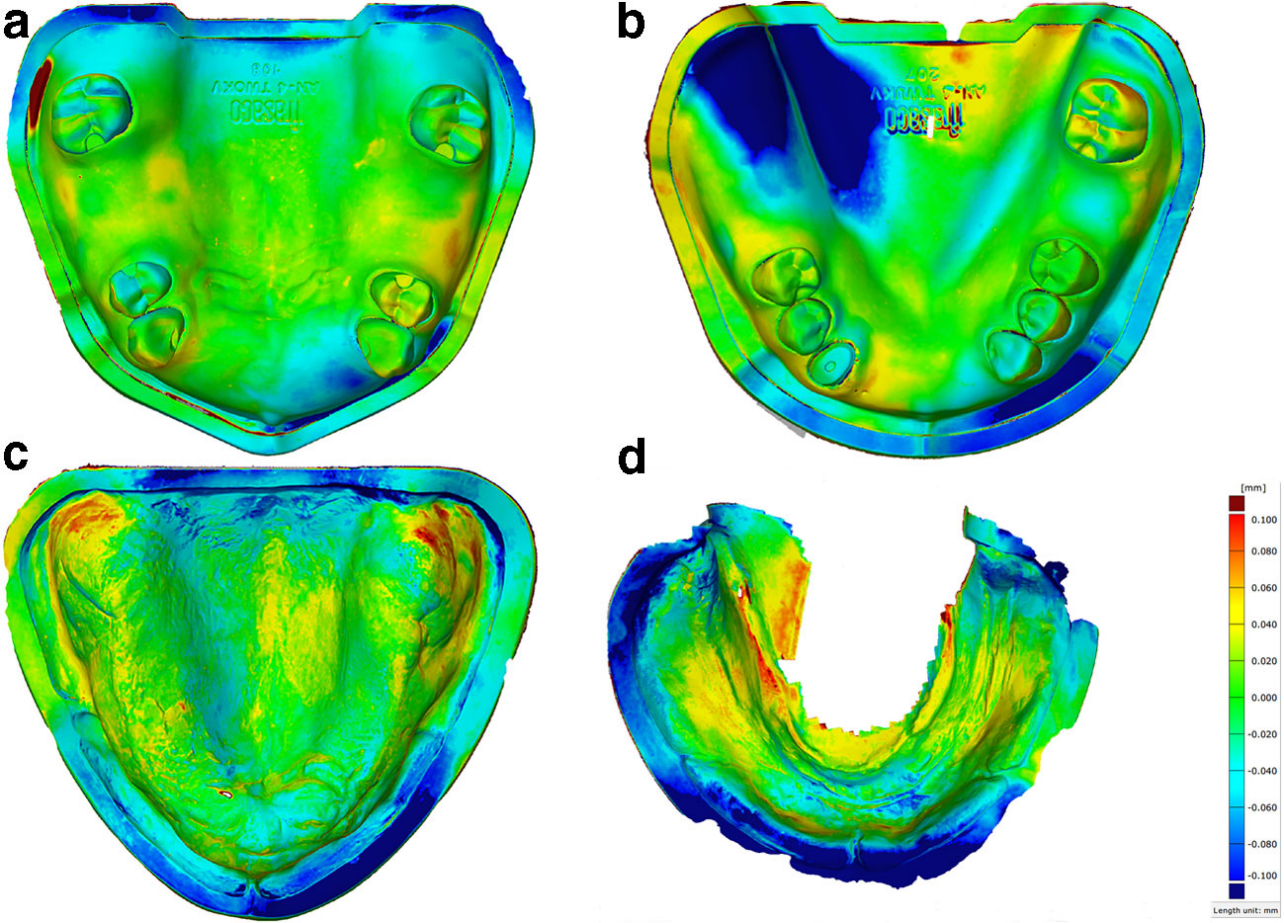
Representative heatmaps after superimposing STL data of intraoral and the reference scans. **a** Partially edentulous maxillary. **b** Partially edentulous mandibular. **c** Completely edentulous maxillary. **d** Completely edentulous mandibular model

The overall median trueness comprising of all digital scans by the two operators was 24.2 μm (IQR 20.7 μm–27.4 μm). The statistical omnibus test yielded a significant influence of the type of model (*p* < 0.0001), the operator (*p* < 0.0001), and of the interaction of the operator and the type of model (*p* < 0.0001) on trueness. Significantly higher trueness was found in the scans of the edentulous mandibular model by the inexperienced operator (*p* = 0.0001). No differences were detected among the other scans (Table 1, Fig. 3). For the scans of the partially edentulous models, the largest deviations were found in the edentulous sites of the anterior maxilla and the right posterior mandible (Fig. 4). The overall median number of surface points was higher in the scans of the inexperienced operator (140,760; IQR 119,753–153,929 vs. 140,544; IQR 124,548–163,047), however, without influence on trueness values (*p* = 0.23).

**Table 1:** Trueness

	Inexperienced (deviations in μm)	Experienced (deviations in μm)	*p* value
Edentulous mandible			
Median (p25, p75)	21.5 (20.4, 24.6)	27.4 (25.6, 30.2)	
Range (min–max)	19.2–25.5	23.7–36.1	*0.0001*
Partial mandible			
Median (p25, p75)	19.5 (18.2, 23.5)	21.0 (17.0, 23.7)	
Range (min–max)	17.0–33.6	14.9–24.5	0.54
Edentulous maxilla			
Median (p25, p75)	35.0 (31.1, 35.5)	29.5 (26.6, 30.7)	
Range (min–max)	23.6–51.0	24.8–37.8	0.11
Partial maxilla			
Median (p25, p75)	22.9 (21.6, 24.0)	21.9 (20.2, 23.5)	0.89
Range (min–max)	16.7–24.4	19.4–24.6	

Median trueness values, interquartile ranges, and minimum and maximum deviations in μm for every cast, and comparison between experienced and inexperienced operator (post hoc pairwise *t* tests)

**Fig. 5:**
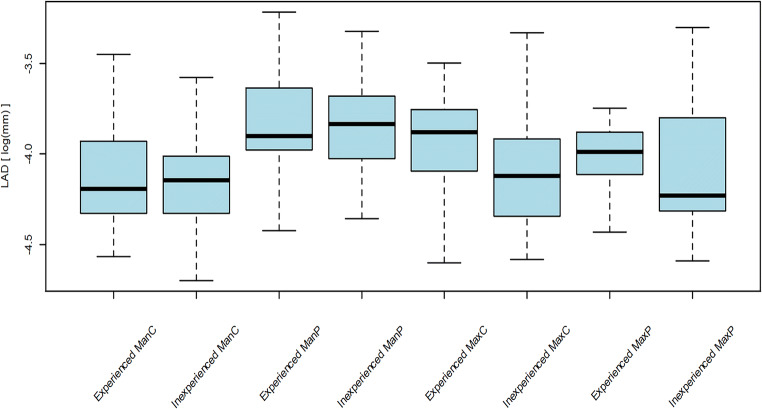
Precision: logarithm of absolute deviations (LADs; *y*-axis), separated for two operators (experienced vs. inexperienced), and different types of models (ManC = mandible completely edentulous, ManP = mandible partially edentulous, MaxC = maxilla completely edentulous, maxilla = partially edentulous)

The overall median precision was 18.3 μm (IQR 14.4–22.1 μm). The statistical omnibus test yielded a significant influence of the type of model (*p* < 0.0001), the operator (*p* = 0.02), and of the interaction of the operator and the type of model (*p* = 0.03) on precision. A significantly higher precision was found for the scans of the edentulous maxillary model by the inexperienced operator (*p* = 0.0004). No differences were detected among the other scans (Table 2, Fig. 5)

**Table 2:** Precision

	Inexperienced (deviations in μm)	Experienced (deviations in μm)	*p* value
Edentulous mandible			
Median (p25, p75)	15.9 (13.2, 18.1)	15.1 (13.2, 19.6)	
Range (min–max)	9.1–27.9	10.2–40.1	0.36
Partial mandible			
Median (p25, p75)	21.6 (17.9, 25.2)	20.2 (18.7, 26.3)	
Range (min–max)	12.8–36.0	12.0–40.1	0.80
Edentulous maxilla			
Median (p25, p75)	16.2 (13.0, 19.9)	20.7 (16.7, 23.4)	
Range (min–max)	10.2–35.7	10.0–30.2	*0.0004*
Partial maxilla			
Median (p25, p75)	14.6 (13.4, - 22.4)	18.5 (16.4, 20.7)	
Range (min–max)	10.2–36.8	11.9–23.6	0.17

Median precision values, interquartile ranges, and minimum and maximum deviations in μm for every cast, and comparison between experienced and inexperienced operator (post hoc pairwise *t* tests)

**Fig. 6:**
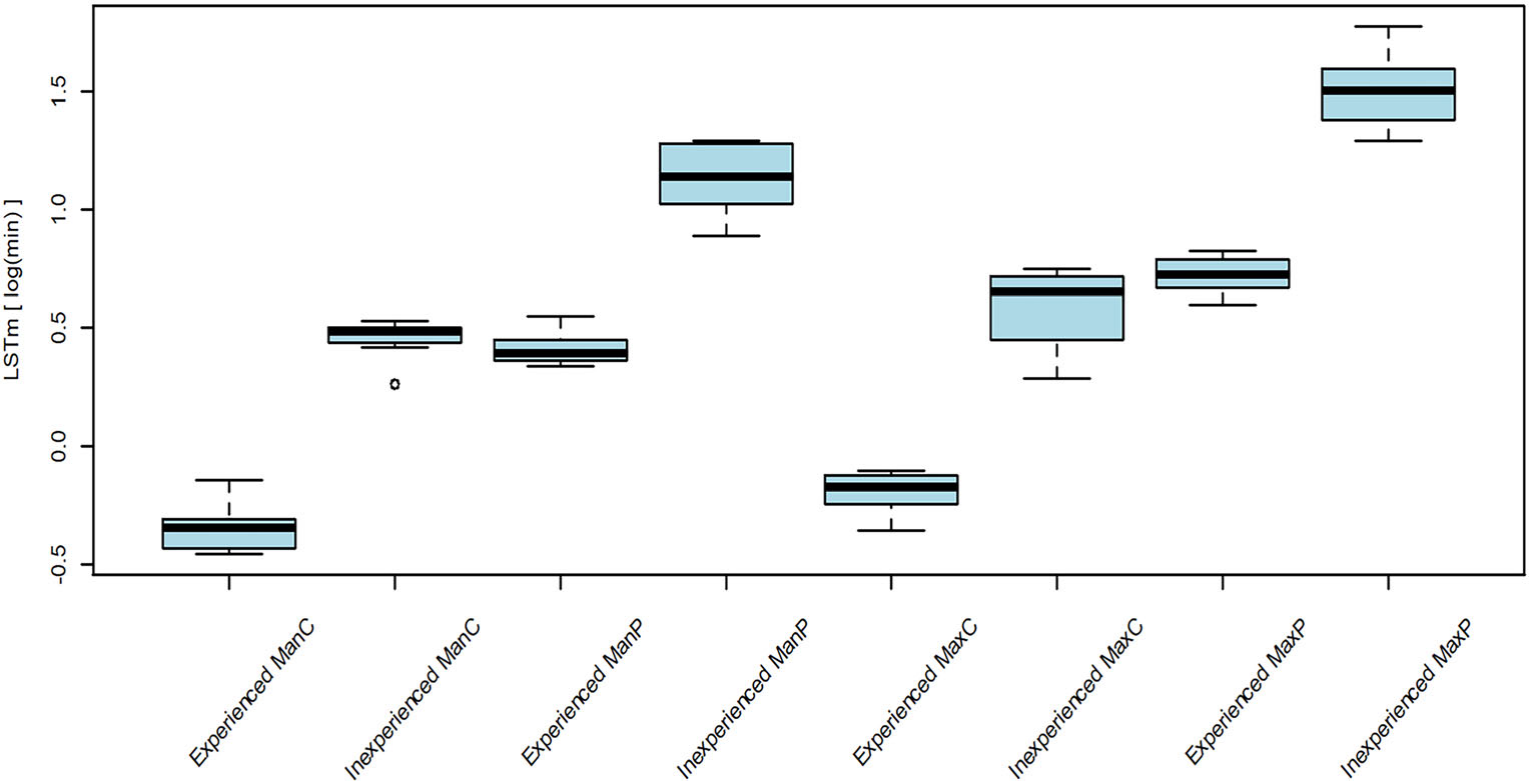
Scan time: logarithm of scan time in (LSTm; *y*-axis), separated for the two operators (experienced vs. inexperienced), and the different types of models (ManC = mandible completely edentulous, ManP = mandible partially edentulous, MaxC = maxilla completely edentulous, maxilla = partially edentulous)

The overall median scan time was 100.5 s (IQR 72.0, 139.2 s). The statistical omnibus test yielded a significant influence of the type of model (*p* < 0.0001) and the operator (*p* < 0.0001) on the scan time. Scans of experienced operator were faster than the scans of inexperienced operator (Table 3, Fig. 6). Longer scan times could be associated with a higher level of trueness (*p* = 0.04)

**Table 3:** Scan time

	Overall (scan time in seconds)	Inexperienced (scan time in seconds)	Experienced (scan time in seconds)	*p* value
Edentulous mandible				
Median (p25, p75)	65.0 (42.8, 97.2)	97.5 (93.8, 98.8)	42.5 (39.8, 44.0)	*< 0.0001*
Range (min–max)	38.0–102.0	78.0–102.0	38.0–52.0	
Partial mandible				
Median (p25, p75)	125.0 (89.5, 185.5)	188.0 (169.2, 213.0)	89.0 (86.2, 93.5)	
Range (min–max)	84.0–218.0	146.0–218.0	84.0–104.0	*< 0.0001*
Edentulous maxilla				
Median (p25, p75)	67.0 (51.2, 113.8)	115.5 (96.2, 123.0)	50.5 (47.2, 52.8)	
Range (min–max)	42.0–127.0	80.0–127.0	42.0–54.0	*< 0.0001*
Partial maxilla				
Median (p25, p75)	177.5 (124.0, 264.5)	270.0 (238.8, 295.2)	124.0 (118.0, 130.8)	
Range (min–max)	109.0–354.0	218.0–354.0	109.0–137.0	*< 0.0001*

Median scan time, interquartile ranges, and minimum and maximum time in seconds; overall and for every cast, as well as separated for the two operators and comparison between the operators (post hoc pairwise *t* tests)

## Discussion

IOS of completely and partially edentulous maxillary and mandibular models resulted in high trueness and precision. The accuracy of the digital scans obtained by the experienced operator was not higher compared to the scans of the inexperienced operator. As a matter of fact, higher trueness was found for the edentulous mandibular and higher precision for the edentulous maxillary model scans of the inexperienced operator. Therefore, in terms of accuracy, the alternative hypothesis had to be rejected. However, the scan time of the experienced operator was shorter, confirming the second part of the alternative hypothesis.

Although no sample size calculation was done, the number of ten scans of each operator-model combination, resulting in 20 scans per model was deemed sufficient when analyzing the accuracy, considering that studies of similar nature analyzed equal or even smaller numbers [12, 24]. In addition, statistical differences were found for trueness, precision, and scan time. However, including only a single IOS experienced and inexperienced operator, respectively, is a limiting factor. All digital scans were performed in a phantom head to simulate the limited space to move the camera intraorally. Other factors, such as patient movement, the presence of saliva or varied light-reflecting due to different kinds of intraoral tissues, which are said to influence the accuracy, were not simulated. However, some recent studies have shown only minor differences of in vivo versus in vitro complete-arch scans with IOS devices, in terms of accuracy and precision [25, 26]. Regarding the digital scans of the non-attached mucosa, which is the major challenge when scanning edentulous sites, a distance of 2 mm away from the mucobuccal fold was chosen simulating the future extension of the denture. As recent studies have proven an improved fit of digitally fabricated RCDs, it might not be necessary to extend the denture borders into the alveolar mucosa to result in adequate stability of an RCD, as it is done in conventionally fabricated RCDs [27, 28]. However, this hypothesis must be confirmed by future studies, as there is no evidence for this theory. Keeping the scan borders 2 mm away from the mucobuccal fold decreased the scanned edentulous area. The decrease in scanned area might be a factor for the high accuracy found in the current study, as an increase in the scanned edentulous area has been reported to influence the accuracy of intraoral scans negatively [15].

Many different techniques analyzing the accuracy of IOS have been reported; however, using reference scan data from an industrial high-precision scanner is still regarded as the gold standard for measuring trueness [4, 29]. Comparing scan data through a best-fit alignment is also a well-accepted methodology, although it has some limitations that have to be taken into account when interpreting the results of the present study. This algorithm attempts to find the superimposition of two surface scans with the minimum difference between all surface points, which can lead to underestimation of the distance between two, particularly selected points [29]. In the present study, it was chosen to apply a local best-fit alignment, only focusing on the surface points of the ROI, simulating the future extension of an RPD or RCD, respectively. As the ROI had to be defined only once for each type of model based on the reference scan data, this technique resulted in a more repeatable superimposition, compared to post-processing of every single scan, in terms of manual trimming of the STL files, and subsequent superimposition. Furthermore, different approaches have been used to describe deviations between digital scan data including root-mean-square(RMS) deviations, average deviations, mean deviations, and absolute deviations [12]. The currently applied technique, the use of absolute amounts of every deviation between two corresponding surface points and subsequently calculating the average, is mathematically similar to the RMS deviations. This similarity between those different techniques enables the comparison of current study results with the studies which used RMS deviations.

The application of only one single intraoral scanner limits the interpretation of the results of present study. A test group with a conventional impression technique was not included, as the accuracy of conventional impressions with a polyvinylsiloxane or polyether respectively, under in vitro conditions has been demonstrated in dentate and edentulous scenarios, before [12, 30, 31]. In those studies, the median deviations of conventional impressions ranged from 7.4 to 39 μm.

The accuracy of IOS in all types of models in the present study was very high. In the current literature, there is only a single study that reports on trueness and precision of the IOS device that was used in this study (Primescan, Sirona) which compared it to different IOS devices [12]. In that study, neither the trueness nor the precision was as high as in the present study, using the same software with a best-fit algorithm for their analyses. Interestingly, they scanned a completely dentate model, in which trueness and precision can be expected to be higher than in an edentulous or partially dentate model. Nevertheless, the Primescan also performed best, of all the applied scanners in that study, but trueness and precision were significantly higher with conventional polyvinylsiloxane impressions. An explanation for the higher accuracy in the present study could be attributed to the newer software version (version 5.0.2), which was not available when the former study was conducted. Compared to other studies reporting on in vitro assessed deviations of polyvinylsiloxane impressions in partially or completely edentulous arches, the median deviations in the present study applying IOS were smaller [32, 33].

The small influence of IOS experience on the accuracy, and even higher trueness and precision found in the edentulous mandibular and maxillary model scans of the IOS-inexperienced operator, were not expected, as the available literature suggests higher accuracy in digital scans of IOS-experienced clinicians [34]. However, it is questionable if the small, but statistically significant difference of trueness and precision between the operators is of any clinical relevance. Considering the results of a recently published study on maxillary complete-arch scans, which reported maximum deviations of 0.3 mm to be clinically relevant, this has at least to be critically scrutinized [35]. The shorter scan times of the IOS-experienced operator were to be expected, as the positive effect of IOS experience on scan time was demonstrated in previous studies [36]. The main reason for the equal trueness and precision values of most of the digital scans by the two operators might be the technological evolution in this new generation IOS device. However, this hypothesis has to be proven by further clinical studies. The longer scan time of the inexperienced operator could be another reason for the higher trueness in the edentulous mandible, and the higher precision in the edentulous maxilla scans, as the statistical analysis showed a direct correlation between longer scan times and higher trueness.

For future research, increasing the sample size for the number of experienced and inexperienced operators would help confirming the results of the current study. Clinical studies evaluating the suitability of IOS for RCD or RPD fabrication under in vivo conditions should also be performed. Controlled trials, comparing clinical- and patient-reported outcomes with dentures, fabricated based on digital scans or conventional impressions would be of particular interest. Furthermore, it would be interesting to investigate the hypothesis, whether denture borders must be extended into the functional zone or due to the improved fit, whether staying in the keratinized attached mucosa might result in adequate stability of a complete denture or not.

## Conclusion

Within the limitations of this in vitro study, it was concluded that the accuracy of IOS in edentulous and partially edentulous models using the tested new generation IOS device (Primescan) was high. The operator’s experience with IOS had only a small influence on the scan accuracy; however, the experienced operator’s scan times were shorter. The intraoral scans obtained with the tested new generation intraoral scanner may be suitable for the fabrication of removable prostheses regardless of clinician’s experience in IOS.
